# Lower Limb Deep Venous Thrombosis After Posterior Spinal Fusion as an Initial Manifestation of Factor V Leiden Mutation in a Pediatric Patient: A Case Report

**DOI:** 10.7759/cureus.57543

**Published:** 2024-04-03

**Authors:** Cristian J Cortes-Nieves, Nicole Ramirez, Aracelis Nieves, Norman Ramírez

**Affiliations:** 1 Orthopedics, University of Puerto Rico, Medical Sciences Campus, San Juan, PRI; 2 Orthopaedic Surgery, Ponce Health Sciences University, Ponce, PRI; 3 Family Medicine, Manati Medical Center, Manati, PRI; 4 Pediatric Orthopedic Surgery, Mayagüez Medical Center, Mayagüez, PRI

**Keywords:** posterior spinal fusion, heterozygous factor v leiden mutation, heterozygous factor v leiden, deep venous thrombosis (dvt), scoliosis surgery

## Abstract

Deep venous thrombosis (DVT) is a serious condition in which a blood clot forms in a deep vein, usually of the lower extremity. In pediatric orthopedic surgery, the incidence of thrombotic events is rare. This is a case presentation of a 12-year-old female patient without previous events or a family history of thrombotic events who underwent a posterior spinal fusion due to severe adolescent idiopathic scoliosis. The patient developed a DVT due to an underlying Factor V Leiden mutation. The purpose of this case report is to create awareness, facilitate the diagnosis and management, and aid in future interventions and clinical outcomes.

## Introduction

Deep venous thrombosis (DVT) is a serious condition in which a blood clot forms in a deep vein, usually of the lower extremity. It presents with symptoms such as pain, swelling, warmth, and changes in skin color [1]. Some risk factors for developing DVT include age, lack of movement, smoking, and surgeries up to three months post-procedure.

In pediatric orthopedic surgery, the incidence of thrombotic events is rare. Children have a degree of physiological protection against DVT after orthopedic surgeries, with an incidence of less than 0.001%. In elective pediatric orthopedic surgeries, incidence has been reported at 0.0515%, on the other hand, none of the patients in a pilot study developed a DVT event after pediatric posterior spinal fusion [2, 3, 4]. Therefore, not giving prophylaxis is the recommendation for a patient without a known underlying risk factor for DVT events.

This is a case presentation of a 12-year-old female patient with menarche at eight years of age, who underwent posterior spinal fusion surgery due to progressive adolescent idiopathic scoliosis. After two months of the surgical procedure, the patient presented with calf pain for which a venous Doppler was performed and confirmed the presence of DVT. Further testing revealed underlying Factor V Leiden mutation. The objective of this case report is to create awareness of potential DVT as an initial manifestation of Factor V Leiden after a posterior spinal fusion in the pediatric population.

## Case presentation

**Figure 1:**
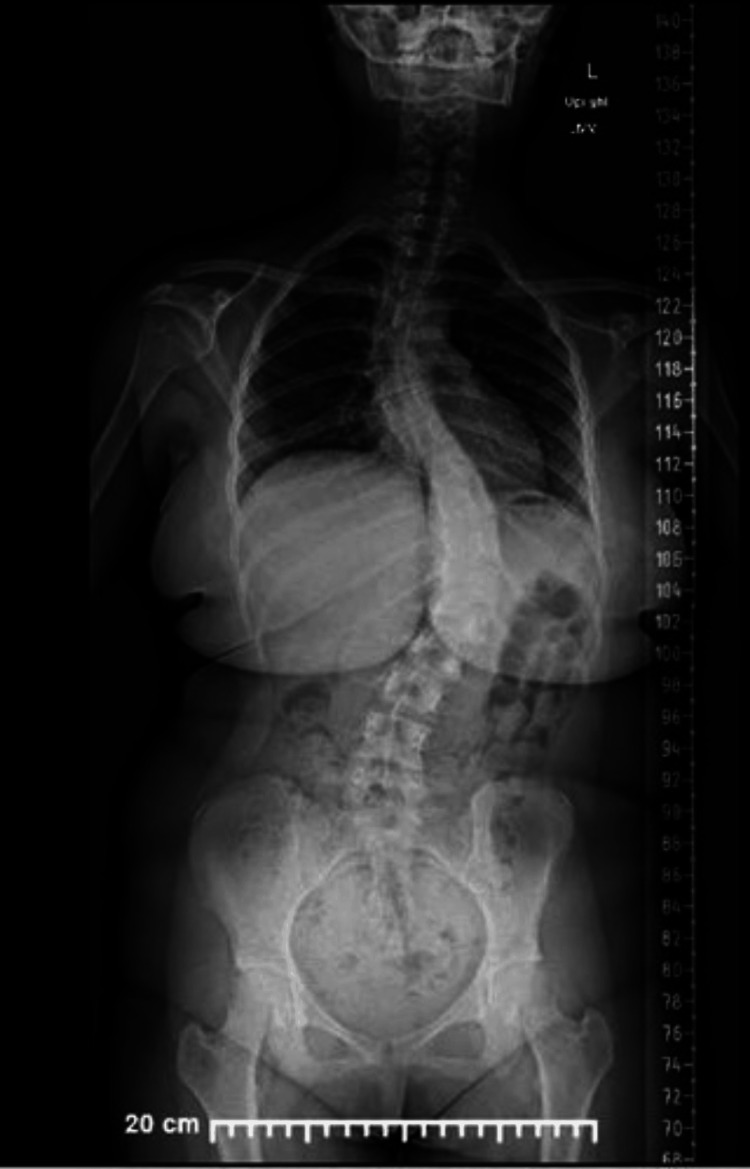
Pre-operative standing posteroanterior imaging. T1-T6: 50°/T7-L3: 60°.

**Figure 2:**
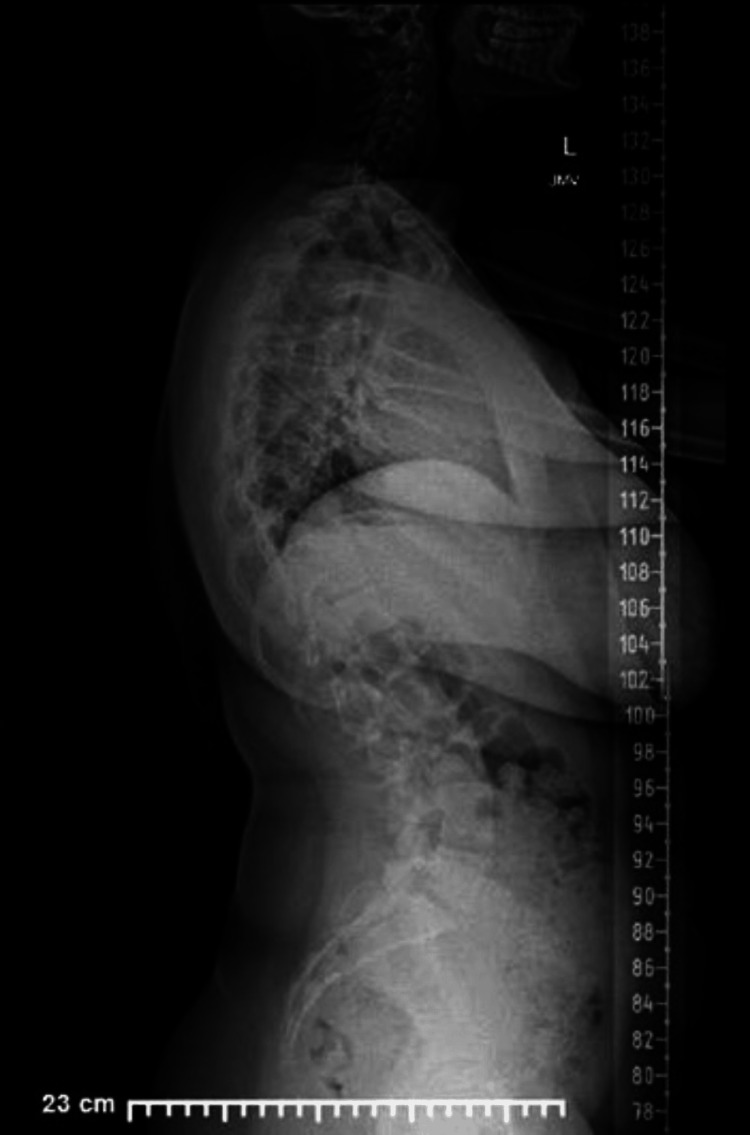
Pre-operative standing sagittal imaging.

This is a case presentation of a 12-year-old female patient with a past medical history of asthma, atopic dermatitis, and allergy to amoxicillin who weighs 59.4 kg, standing 1.5 m tall (body mass index: 25.6 kg/m^2^, obesity). Her menarche was at eight years of age with a regular pattern. The patient has no prior surgical history. Family history is relevant for hypertension, cancer, and thyroid problems. The patient was admitted for a posterior spinal fusion due to severe adolescent idiopathic scoliosis of 35° from thoracic level number 1 to thoracic level number 6 and 60° from thoracic level number 7 to lumbar level number 3 (Figures 1, 2). The pre-operative laboratory work-up demonstrated: white blood cells, hemoglobin, hematocrit, platelets, prothrombin time, and partial thromboplastin time all within normal range. International normalized ratio mildly increased (Table 1).

**Figure 3:**
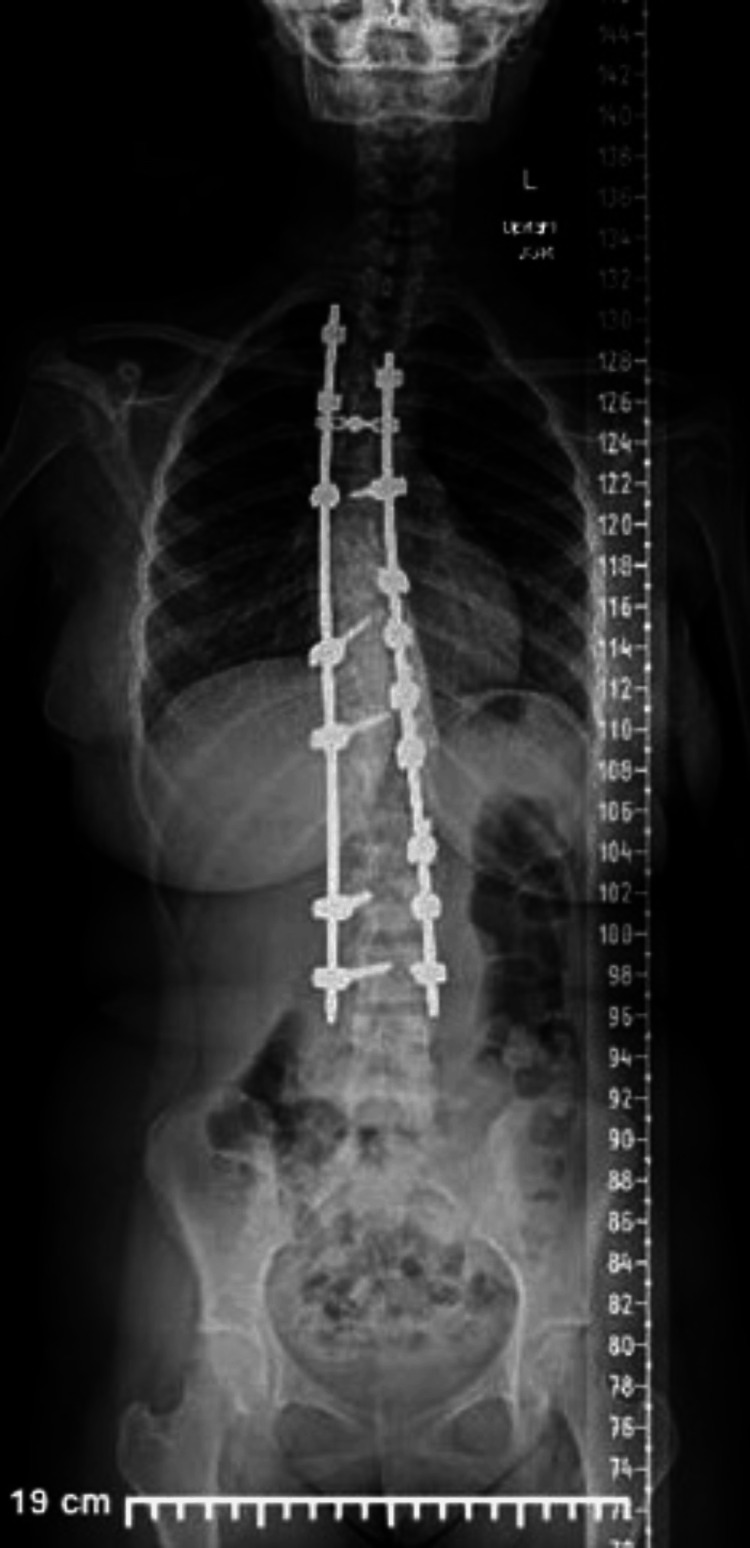
Post-operative standing posteroanterior imaging.

**Figure 4:**
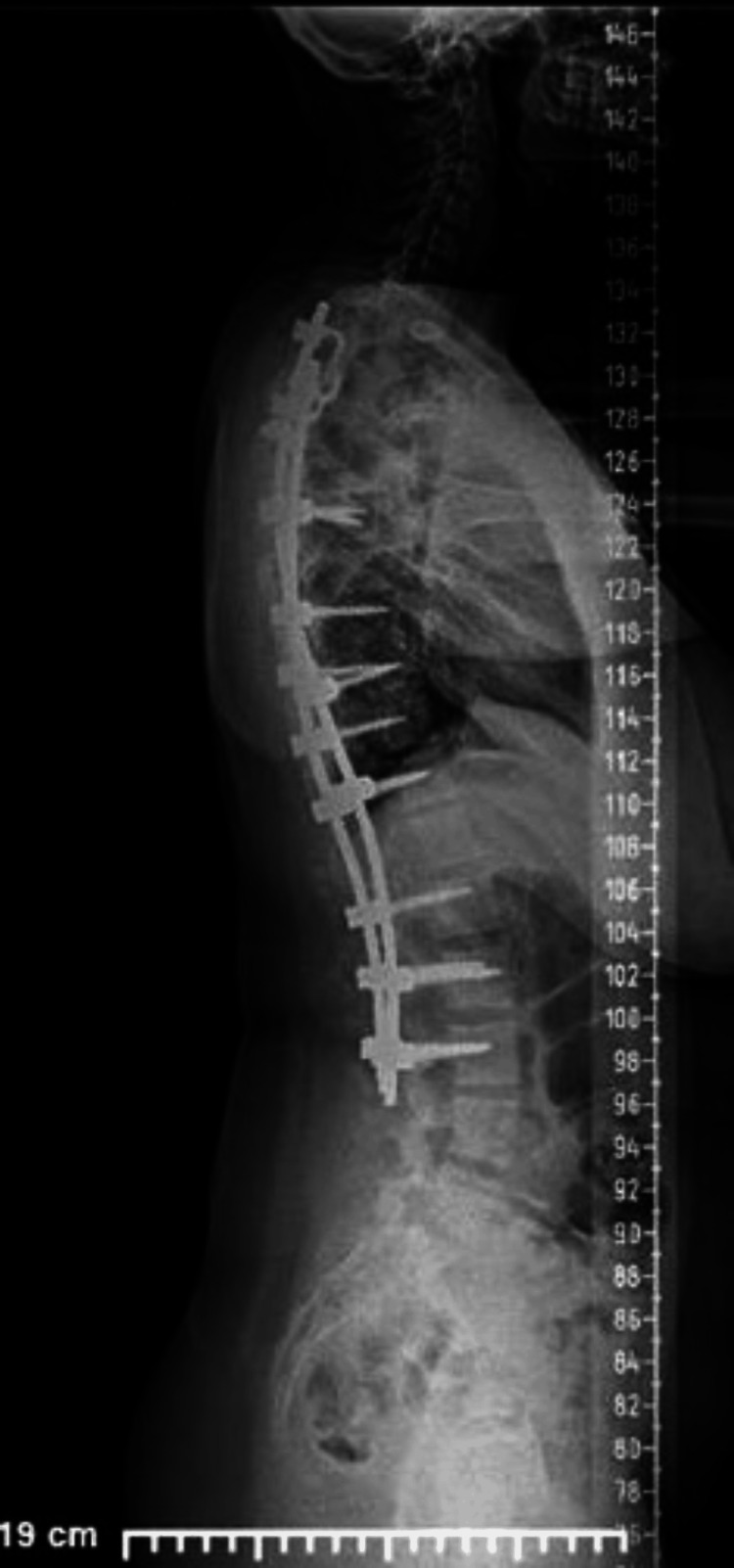
Post-operative standing sagittal imaging.

The surgical time was five hours and 30 minutes, from thoracic level 3 to lumbar level 3 (Figures 3, 4). The estimated blood loss was 300 mL. No intra-operative or post-operative complications, such as severe blood loss or neurological deficits, were documented. As per protocol, the patient was out of bed and ambulating at 24 hours post-procedure. The post-operative laboratory work-up showed, at 36 hours post-procedure, a hemoglobin level of 9.2 g/dL and a hematocrit level of 28.4%. The patient was discharged home, ambulating without acute distress, on the third day post-procedure.

The patient had an unremarkable post-operative period until two months after the surgical procedure, when she developed left antero-medial thigh pain with mild swelling, along the L3 dermatome without calf pain or involvement. She denied a history of trauma, fever, chills, shortness of breath, bleeding, nausea, vomiting, or recent prolonged travel. The pain was managed with gabapentin, as it was thought to be due to post-operative radiculopathy. A thoracic and lumbar computed tomography scan was ordered to ensure proper placement of pedicle screws, which demonstrated appropriate anatomical positioning. One week later, the patient presented with an intensification of pain with progression to the popliteal fossa and calf. Physical examination demonstrated the left calf warm with swelling and tenderness to palpation. There was a measurement discrepancy of 37 cm on the left calf and 32 cm on the right calf. The Homan’s test was compatible with a DVT. D-dimer testing was markedly increased (Table 1). Due to findings suggestive of possible DVT, an iliocaval venous Doppler was performed, which demonstrated evidence of deep venous thrombosis with partial compression of the left common iliac vein and external iliac vein. DVT diagnosis was confirmed, and low molecular weight heparin (Enoxaparin) 60 mg SC q12h was administered throughout the hospital stay. The patient was immediately consulted to hematology pediatric services.

Pro-thrombotic work-up was ordered, which included antithrombin III, protein S, protein C activity, factor VIII activity, cardiolipin immunoglobulin G & M, lupus anticoagulant, prothrombin mutation, and factor V Leiden mutation. Results demonstrated: antithrombin III activity within normal range, protein S activity decreased, protein C activity decreased, factor VIII activity increased, cardiolipin IgG within normal range, cardiolipin IgM within normal range, lupus anticoagulant negative, and prothrombin mutation negative. However, the Factor V Leiden heterozygous mutation was positive (Table 1). Hematology services planned to continue anticoagulation for three months and repeat imaging to assess for clot resolution. On repeat imaging examination, partial compressibility with evident intraluminal echoes and re-tunneling in the left common femoral vein was observed. These findings were suggestive of chronic DVT. Observation and direct oral anticoagulant (Apixaban) 5 mg BID PO was recommended by the periphero-vascular pediatric surgical service due to asymptomatic clinical findings.

**Table 1:** Laboratory work-up with laboratory parameters and reference ranges. WBC: white blood cells, Hgb: hemoglobin, Hct: hematocrit, Plt: platelets, PT: prothrombin time, PTT: partial thromboplastin time, INR: international normalized ratio, MPL: microgram of IgM phospholipid units.

Laboratory parameters	Patient values	Reference ranges
WBC (n/µL)	5.56 × 10^3^	4 × 10^3^ to 10 × 10^3^
Hgb (g/dL)	12.6	11.5–15.5
Hct (%)	37.2	35.5-44.9
Plt (µL)	289 × 10^3^	150 × 10^3^ to 400 × 10^3^
PT (s)	13.6	12.3–15.1
PTT (s)	28.4	29.0–43.0
INR	1.13	0.8–1.11
D-dimer (mg/L)	4.22	<0.50
Antithrombin III (%)	108	80–120
Protein S activity (%)	55	60–150
Protein C activity (%)	60	65–135
Factor VIII activity (%)	179	50–150
Cardiolipin IgG (U/mL)	2.00	<15
Cardiolipin IgM (U/mL)	<0.20	<12.5
Lupus anticoagulant (MPL)	Negative	20–39
Prothrombin mutation	Negative	Negative
Factor V Leiden mutation	Positive	Negative

## Discussion

Postoperative radiculopathy pain after a posterior spinal fusion in an adolescent patient can present as a neurological complication manifesting as extremity weakness or pain, due to spinal cord compression [5]. It has been shown that radiculopathy can also present as an expression of iatrogenic causes, such as mispositioning of pedicle screws [6, 7]. Mispositioning of screws was evaluated with a computed tomography scan and was excluded as a diagnosis. The patient was managed initially as probable radiculopathy; however, a week later, she presented with clinical findings of a DVT, which was then confirmed through venous Doppler and D-dimer levels. This brings to light another possibility for postoperative lower extremity pain in an adolescent patient, which is the occurrence of a DVT.

DVT is one of the clinical manifestations of venous thromboembolism. The leading theory delineating the pathogenesis of DVT is the Virchow triad, which proposes that it is the result of alteration of blood flow, vascular endothelial injury, and alterations in the constituents of the blood. It has been seen that in patients with venous thrombosis present, approximately 56% have three or more risk factors such as surgery, hospital admission, malignancy, infection in the last three months, more than 48 hours of immobilization, or current hospitalization for the development of DVT [8].

Surgery is a known risk factor for developing a DVT in the adult population; however, in a pediatric population, DVT is rare and highly associated with hematological underlying pathology. In a pilot study of DVT screening after idiopathic scoliosis in children, of the patients seen, none developed a DVT [4]. Additionally, a study performed to evaluate the incidence of DVT in children after elective orthopedic surgery demonstrated an incidence of 5.1 per 10,000 patients, with a total of 74 cases of which 41 occurred after discharge. Among the varied etiology for the occurrence of DVT in children, we can find Factor V Leiden mutation. The pro-thrombotic work-up on the patient demonstrated mildly decreased protein S and protein C activity, elevated factor VIII activity, and a positive Factor V Leiden heterozygous mutation. It is pertinent and of utmost importance to bring to clinical consciousness the possibility of an underlying Factor V Leiden mutation in this population.

Factor V Leiden is an autosomal dominant genetic condition with incomplete penetrance. Most presentations are heterozygous, while homozygous or pseudo-homozygous have been shown to be at an increased risk of DVT [9]. On the other hand, patients with the heterozygous mutation have in their plasma normal Factor V in addition to mutated Factor V, giving them a degree of protection against thrombosis. The resulting pathology is derived from a single-point mutation in the Factor V gene, resulting in a change of arginine for glutamine at amino acid 506. This amino acid change degenerates the cleavage site for activated protein C, a protease that degrades Factor V. Therefore, it develops a “Protein C resistance,” leading to increased levels of activated Factor VIII and activated Factor V, increasing the risk of thromboembolism. Nonetheless, only a small percentage of individuals with the Factor V Leiden mutation will develop a DVT in their lifetime.

DVT is the most common manifestation of Factor V Leiden mutation. The first occurrence of a DVT is not an indication to test for the mutation; however, in an adolescent patient, it is pertinent to search for underlying causes, and it is therefore indicated to test. Additionally, it is common for a patient with a Factor V Leiden mutation to have a concomitant prothrombin G20210A mutation, which greatly increases the risk for DVT. Different studies have evaluated the risk of DVT recurrence in Factor V Leiden heterozygous mutation patients and have demonstrated odds of 1.36 [1]. The management of patients with Factor V Leiden heterozygous mutation is indifferent for the general population, which should be with direct oral anticoagulants. Long-term anticoagulation should not be offered unless there is a high risk of recurrence or unprovoked, life-threatening DVT because treatment after three months has risks of bleeding that outweigh the risk of recurrence [10]. Lastly, patients who are found to have the Factor V Leiden mutation should receive prophylactic anticoagulation for surgeries.

Vitamin K antagonists and oral anticoagulation are the most effective long-term treatments for preventing complications such as thromboembolisms [11]. Nonetheless, direct oral anticoagulants are increasingly being used in a variety of conditions and are currently gaining popularity, quickly becoming the mainstay treatment for acute DVT for a role in prolonged treatment due to a decreased risk of bleeding [12]. The role of direct oral anticoagulants in Factor V Leiden mutation comes into play through inhibition of Factor Xa (thrombin), disabling its capacity to continue the coagulation cascade and thereby decreasing the risk of DVT occurrence. It has been shown that it is not inferior to low-molecular-weight heparin (LMWH) and has advantages over vitamin K antagonists such as predictable pharmacokinetics, rapid onset of action, and ease of administration without monitoring [1, 10, 12]. Moreover, there is a significant reduction in the two-year risk of recurrence of a DVT when compared to vitamin K antagonists [13].

## Conclusions

Lower extremity pain after posterior spinal fusion for scoliosis in pediatric patients is often associated with radiculopathy, which could be iatrogenic. Another possibility is a lower extremity DVT. The development of a DVT is rare after spinal surgery in children, but it is important to consider the potential of occurrence due to underlying coagulopathies such as Factor V Leiden mutation in a patient without prior events. In the occurrence of a DVT, management should include the use of direct oral anticoagulants, which could reduce recurrence. Although prophylaxis is not warranted on a patient without a history of DVT events, maintaining it as a possible incident can facilitate the diagnosis and management and aid in future interventions and clinical outcomes.
